# Molecular Epidemiology and Antifungal Susceptibility of *Candida glabrata* in China (August 2009 to July 2014): A Multi-Center Study

**DOI:** 10.3389/fmicb.2017.00880

**Published:** 2017-05-23

**Authors:** Xin Hou, Meng Xiao, Sharon C.-A. Chen, Fanrong Kong, He Wang, Yun-Zhuo Chu, Mei Kang, Zi-Yong Sun, Zhi-Dong Hu, Ruo-Yu Li, Juan Lu, Kang Liao, Tie-Shi Hu, Yu-Xing Ni, Gui-Ling Zou, Ge Zhang, Xin Fan, Yu-Pei Zhao, Ying-Chun Xu

**Affiliations:** ^1^Department of Clinical Laboratory, Peking Union Medical College Hospital, Chinese Academy of Medical SciencesBeijing, China; ^2^Graduate School, Peking Union Medical College, Chinese Academy of Medical SciencesBeijing, China; ^3^Beijing Key Laboratory for Mechanisms Research and Precision Diagnosis of Invasive Fungal DiseasesBeijing, China; ^4^Centre for Infectious Diseases and Microbiology Laboratory Services, Westmead Hospital, University of SydneySydney, NSW, Australia; ^5^Department of Clinical Laboratory, The First Hospital of China Medical UniversityShenyang, China; ^6^Department of Laboratory Medicine, West China Hospital, Sichuan UniversityChengdu, China; ^7^Department of Clinical Laboratory, Tongji Hospital, Tongji Medical College, Huazhong University of Science and TechnologyWuhan, China; ^8^Department of Clinical Laboratory, Tianjin Medical University General HospitalTianjin, China; ^9^Department of Clinical Laboratory, Peking University First HospitalBeijing, China; ^10^Department of Clinical Laboratory, The First Affiliated Hospital of Harbin Medical UniversityHarbin, China; ^11^Department of Clinical Laboratory, The First Affiliated Hospital of Sun Yat-Sen UniversityGuangzhou, China; ^12^Department of Clinical Laboratory, The People's Hospital of Liaoning ProvinceShenyang, China; ^13^Department of Clinical Microbiology and Infection Control, Ruijin Hospital Affiliated to School of Medicine, Shanghai Jiaotong UniversityShanghai, China; ^14^Department of Clinical Laboratory, The Fourth Affiliated Hospital of Harbin Medical UniversityHarbin, China; ^15^Department of General Surgery, Peking Union Medical College Hospital, Chinese Academy of Medical SciencesBeijing, China

**Keywords:** *Candida glabrata*, multilocus sequence typing (MLST), microsatellite genotyping, antifungal susceptibility, China

## Abstract

*Candida glabrata* is an increasingly important cause of invasive candidiasis. In China, relatively little is known of the molecular epidemiology of *C. glabrata* and of its antifungal susceptibility patterns. Here we studied 411 non-duplicate *C. glabrata* isolates from 411 patients at 11 hospitals participating in the National China Hospital Invasive Fungal Surveillance Net program (CHIF-NET; 2010-2014). Genotyping was performed using multilocus sequence typing (MLST) employing six genetic loci and by microsatellite analysis. Antifungal susceptibility testing was performed using Sensititre YeastOne™ YO10 methodology. Of 411 isolates, 35 sequence types (ST) were identified by MLST and 79 different genotypes by microsatellite typing; the latter had higher discriminatory power than MLST in the molecular typing of *C. glabrata*. Using MLST, ST7 and ST3 were the most common STs (66.4 and 9.5% of all isolates, respectively) with 24 novel STs identified; the most common microsatellite types were T25 (30.4% of all isolates) and T31 (12.4%). Resistance to fluconazole (MIC > 32 μg/mL) was seen in 16.5% (68/411) of isolates whilst MICs of >0.5 μg/mL for voriconazole, >2 μg/mL for itraconazole and >2 μg/mL for posaconazole were seen for 28.7, 6.8, and 7.3% of isolates, respectively; 14.8% of all isolates cross-resistant/non-wide-type to fluconazole and voriconazole. Fluconazole resistant rates increased 3-fold over the 5-year period whilst that of isolates with non-WT MICs to voriconazole, 7-fold. All echinocandins exhibited >99% susceptibility rates against all isolates but notably one isolate exhibited multi-drug resistance to the azoles and echinocandins. The study has provided a global picture of the molecular epidemiology and drug resistance rates of *C. glabrata* in China during the period of the study.

## Introduction

*Candida* species are the most common opportunistic fungal pathogens in debilitated or immunecompromised hosts with high rates of mortality (up to 40%) (Hajjeh et al., 2004; Wisplinghoff et al., 2004; Kullberg and Arendrup, 2015; Pappas et al., 2016). Although the majority of cases of invasive candidiasis (IC) are attributed to *Candida albicans*, globally, there are increasing rates of infection by non-*C. albicans* species (Kullberg and Arendrup, 2015; Xiao et al., 2015; Pappas et al., 2016). The prevalence of *Candida glabrata* infections, in particular, has increased in the last decade, and this species is now the second most common cause of candidemia in the USA, accounting for up to one-third of cases of fungemia (Pfaller et al., 2001; Guinea, 2014). Data from the China Hospital Invasive Fungal Surveillance Net (CHIF-NET) study have indicated that *C. glabrata* species complex was the third most common non-*C. albicans* species in China (Wang et al., 2012; Xiao et al., 2015).

*Candida glabrata* complex comprises *C. glabrata* sensu stricto but also encompasses the cryptic species *Candida bracarensis* and *Candida nivariensis* (Hou et al., 2017). Yet within *C. glarbata* sensus strcito *per se*, intra-species delineation is useful, not only for molecular epidemiological studies but for investigation of biological niches and determining the route of infection transmission. Several molecular typing methods, e.g., pulse field gel electrophoresis (PFGE), multilocus sequence typing (MLST) assays and microsatellite analysis have been established to determine genetic relatedness of *C. glabrata* (Dodgson et al., 2003; Foulet et al., 2005; Lin et al., 2007; Brisse et al., 2009; Enache-Angoulvant et al., 2010; Abbes et al., 2012). Of these, MLST is a highly discriminatory tool that can be standardized to allow objective comparison of results between centers. The use of a set of six gene fragments of *C. glabrata (FKS, LEU2, NMT1, TRP1, UGP1*, and *URA3*) is recommended (http://cglabrata.mlst.net/) (Dodgson et al., 2003). Microsatellite marker analysis is another rapid, reliable technique with several multilocus microsatellite systems proposed (Foulet et al., 2005; Brisse et al., 2009; Enache-Angoulvant et al., 2010; Abbes et al., 2012).

Data on the susceptibilities to antifungal agents are also important to guide best practice empirical antifungal therapy in patients with suspected *C. glabrata* IC (Yapar, 2014). *C. glabrata* is known to exhibit reduced susceptibility or resistance to fluconazole and the other azoles (Pfaller et al., 2004; Delliere et al., 2016). Further, resistance to the echinocandins (up to 10% in some centers), as well as of echinocandin and azole co-resistance is of growing concern in the USA (Pfaller et al., 2012; Alexander et al., 2013; Pham et al., 2014). In Europe, the prevalence of echinocandin resistance amongst *C. glabrata* isolates is low (<3%) (Delliere et al., 2016). In China, data on azole and echinocandin resistance are relatively sparse (Xiao et al., 2015).

In the present study, we investigated the nationwide molecular epidemiology and *in vitro* antifungal susceptibility of *C. glabrata* sensu stricto isolates causing IC in China during 2010–2014. In this study, MLST genotyping as well microsatellite analysis techniques were employed given their high discriminatory utility.

## Materials and methods

### Ethics statement

The study was approved by the Human Research Ethics Committee of Peking Union Medical College Hospital (No. S-263). Written informed consent were obtained from all patients in the study for permission to study the isolates cultured from them for scientific research.

### Yeast isolates and identification

**Figure 1:**
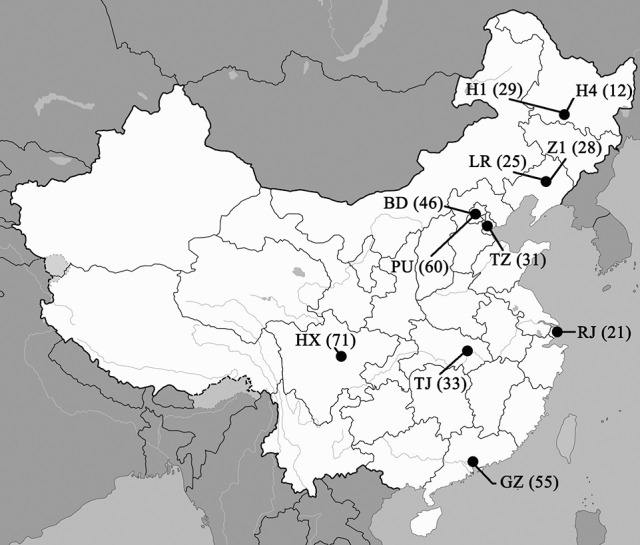
**Geographic distribution of the 11 study centers involved in this study and number of isolates collected in each center (shown in brackets)**. Hospital codes: BD, Peking University First Hospital; GZ, The First Affiliated Hospital of Sun Yat-Sen University; H1, The First Affiliated Hospital of Harbin Medical University; H4, the Fourth Affiliated Hospital of Harbin Medical University; HX, West China Hospital; LR, The People's Hospital of Liaoning Province; PU, Peking Union Medical College Hospital; RJ, Ruijin Hospital, School of Medicine, Shanghai Jiaotong University; TJ, Tongji Hospital; TZ, Tianjin Medical University General Hospital; Z1, The First Hospital of China Medical University.

*Candida glabrata* isolates were collected prospectively over the 5-year study period from patients enrolled in the CHIF-NET study, a laboratory-based, national multicenter surveillance program conducted during August 2009 to July 2014. Only unique isolates i.e., only one strain per patient, were studied (Wang et al., 2012). A total of 411 clinical isolates from 411 patients in 11 hospitals (eight provinces) across China were analyzed (Figure 1, see Acknowledgments for participating hospitals). Isolates were identified as *C. glabrata* by a previously-established algorithm incorporating matrix-assisted laser desorption ionization-time of flight mass spectrometry (MALDI-TOF MS) (Vitek MS, bioMérieux, Marcy l'Etoile, France) supplemented with rDNA internal transcribed spacer (ITS) sequencing (Zhang et al., 2014). Only *C. glabrata* sensu stricto isolates were studied and the confidence value of Vitek MS was ≥90%. For each isolate, a minimum of five colonies were picked from a pure culture together and stored at −80°C in separate vials until use. Early experiments showed that the MLST and microsatellite results were identical for each of these five colonies (data not shown) and previous study described mixture of genotype in one out of 101 (1/101, 1%) isolates (Delliere et al., 2016).

### Multilocus sequence typing (MLST)

Total DNA was extracted from pure cultures as described previously (Wang et al., 2012). Briefly, six housekeeping gene loci (*FKS, LEU2, NMT1, TRP1, UGP1*, and *URA3*) were studied (Dodgson et al., 2003). The PCR products were sequenced in both directions using the DNA analyzer ABI 3730XL system (Applied Biosystems, Foster City, CA). Nucleotide sequences were analyzed manually to ensure high quality sequences, and then queried against the *C. glabrata* MLST database (http://cglabrata.mlst.net) to assign alleles for each locus. The sequence type (ST) was then defined according to isolates' allelic profiles. Novel allele types in each novel ST were confirmed twice by sequencing in both directions.

### Microsatellite analysis

Yeast isolates were genotyped using six highly polymorphic microsatellite markers namely *RPM2, ERG3, MTI, GLM4, GLM5*, and *GLM6*, chosen for their high discriminatory power (Abbes et al., 2012). The forward primers were labeled with carboxyfluorescein (FAM), hexachlorofluorescein (HEX), faststart universal SYBR Green Master (ROX), and carbosytetramethylrhodamine (TAMRA). Amplification reactions were performed as previously reported (Abbes et al., 2012). Following PCR, amplicons were sized by capillary electrophoresis on an ABI 3730XL DNA Analyzer (Applied Biosystems,) coupled with GeneMarker v1.8 software (SoftGenetics LLC, State College, PA, USA). Allele sizes were scored with respect to the GeneScan™ 500 LIZ® Size Standard (Applied Biosystems).

### Antifungal susceptibility tests

Susceptibility tests were performed by using the Sensititre YeastOne™ YO10 (SYO) methodology (Thermo Scientific, Cleveland, OH, USA). *Candida parapsilosis* ATCC 22019 and *Candida krusei* ATCC 6258 were quality control strains. MIC values were interpreted according to CLSI M27-S4 guidelines for fluconazole and echinocandins (Clinical and Laboratory Standards Institute, 2012). The breakpoint for resistance to fluconazole is MIC > 32 μg/ml, to anidulafungin and caspofungin is MIC ≥ 0.5 μg/ml and to micafungin is MIC ≥ 0.25 μg/ml (Clinical and Laboratory Standards Institute, 2012). Where there were no clinical break points (for voriconazole, itraconazole, posaconazole, 5-flucytosine and amphotericin B), species-specific epidemiological cut-off values (ECVs) were used to define isolates as wide-type (WT) or non-WT. The ECV for non-WT to voriconazole and 5-flucytosine is MIC > 0.5 μg/ml and to itraconazole, posaconazole and amphotericin B is MIC > 2 μg/ml (Huang et al., 2014).

### Statistical analysis

The genetic relationships of the isolates were determined by cluster analysis using the minimum-spanning tree available in the BioNumerics software v 6.5 (Applied Maths). To compare the discriminatory power of different molecular methods, we used an index of discrimination (D) based on Simpson's index of diversity and confidence intervals for D were determined by a method described previously by Grundmann et al. (2001).

Data were analyzed with IBM SPSS software (version 22.0; IBM SPSS Inc., New York, USA). Categorical variables were compared using the χ^2^ test. A *P* value of 0.05 was considered significant.

## Results

### Source of isolates, demographics and body site of isolation

Of 411 isolates, 163 (39.7%) were from patients admitted in the Intensive care unit (ICU), 29.7% (122/411) from patients in the Surgery Department, 18.2% (75/411) from the Medical Department, 4.9% (20/411) from the Emergency Department and 7.5% (31/411) from other departments. The average age of the patients (241 males and 170 females) was 60 ± 18.4 years (range 0-96). Nearly one-half (200/411; 48.7%) of the isolates were obtained from blood cultures, 23.1% (95/411) were from ascitic fluid (Tables S1, S2), 5.1% (21/411) from pus and 4.6% (19/411) from venous catheter. The remaining isolates (*N* = 76) were obtained from bile, pleural fluid and other sterile body fluids.

### MLST and microsatellite analysis

**Figure 2:**
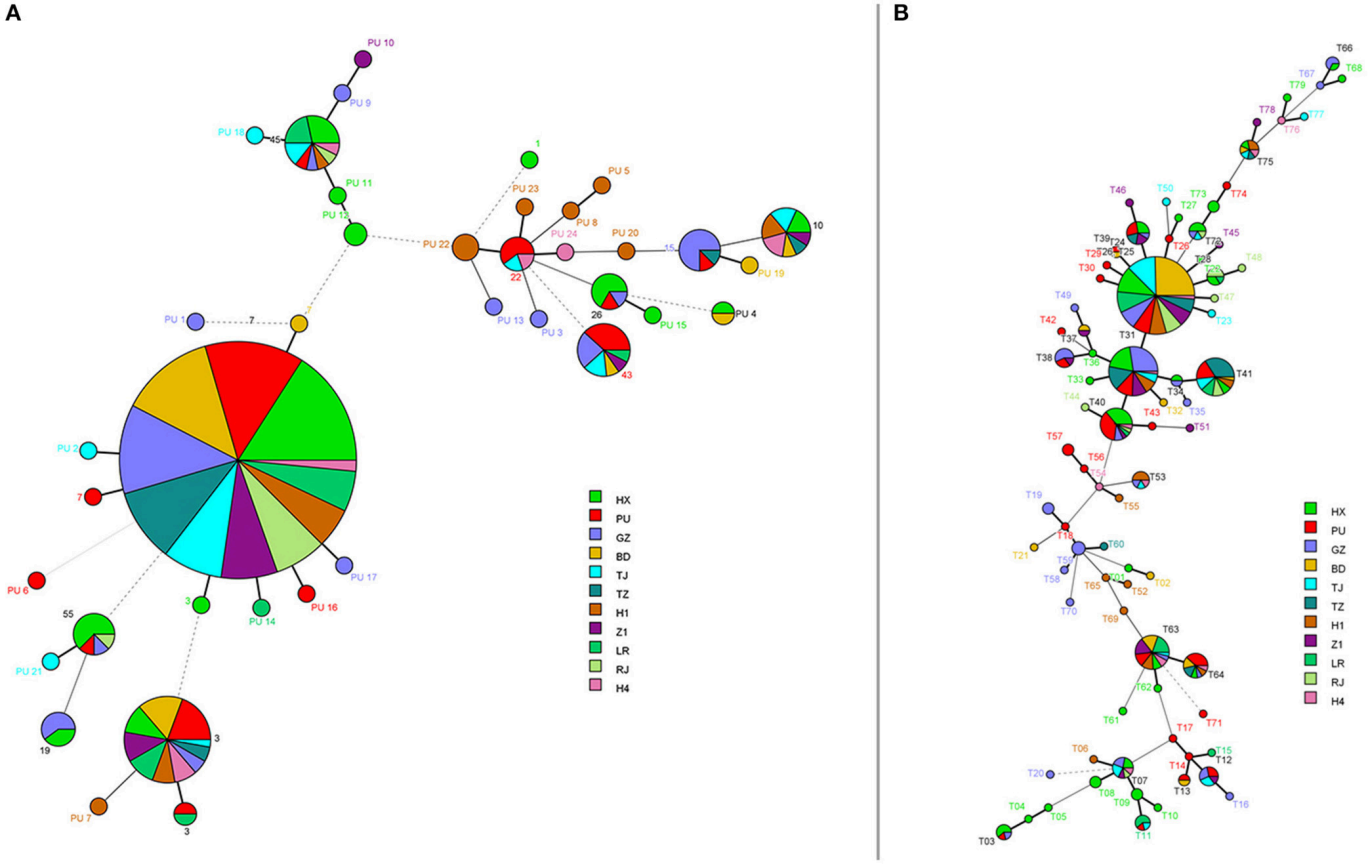
**Minimum spanning tree analysis based on allelic profiles of multilocus sequence typing (MLST) and microsatellites genotypes**. The different circle colors represent different hospitals. In **(A)**, each circle corresponds to a MLST ST. For **(B)**, each circle corresponds to a microsatellite genotype.

In general, MLST analysis revealed a low degree of genetic diversity within *C. glabrata* although the six-locus based MLST scheme showed a large number of STs overall—it allowed for the differentiation of 35 sequence types (STs) among 411 isolates, including 24 novel STs (PU 1-PU 24). The commonest ST, however, was ST7 (273/411 or 66.4% of isolates), where this ST was the predominant ST across all 11 hospitals, followed by ST3 (*n* = 39; 9.5%) (Figure 2A). The diversity index varied from 0.33 for *UGP1* to 0.53 for *NMT1/TRP1*. The D value from all 6 markers was 0.55 (95% confidence interval: 0.49–0.61; Table 1).

**Table 1:** **Discriminatory power of the typing methods used in this study**.

**Methods/Marker**	**No. of genotypes/Different allels**	**Index of discrimination**
**MLST**	**35**	**0.55**
FKS	9	0.5
LEU2	9	0.47
NMT1	14	0.53
TRP1	16	0.53
UGP1	7	0.33
URA3	11	0.49
**Microsatellite**	**79**	**0.88**
RPM2	4	0.46
MTI	8	0.61
ERG3	14	0.52
GLM4	13	0.71
GLM5	14	0.7
GLM6	10	0.53

*MLST, multilocus sequence typing*.

On analysis of ST according to body site of isolation, the majority of isolates from blood (136/200, 68%) and ascitic fluid (60/95, 63.2%), but also all from other specimen types were also of the ST7 type (*n* = 273, 66.4%) followed by ST3 (*n* = 39, 9.5%) (Table S1). Both these STs were identified in 10 of the 11 hospitals (Figure 2A) and further, were the predominant ST in all of fluconazole susceptible-dose dependent (S-DD; *n* = 343) and fluconazole-resistant isolates (*n* = 68) (Figures S1, S2). A further 10 STs, each encompassing two to 14 isolates, were also detected, whereas the remaining 23 STs comprised one isolate each (Table S1).

**Figure 3:**
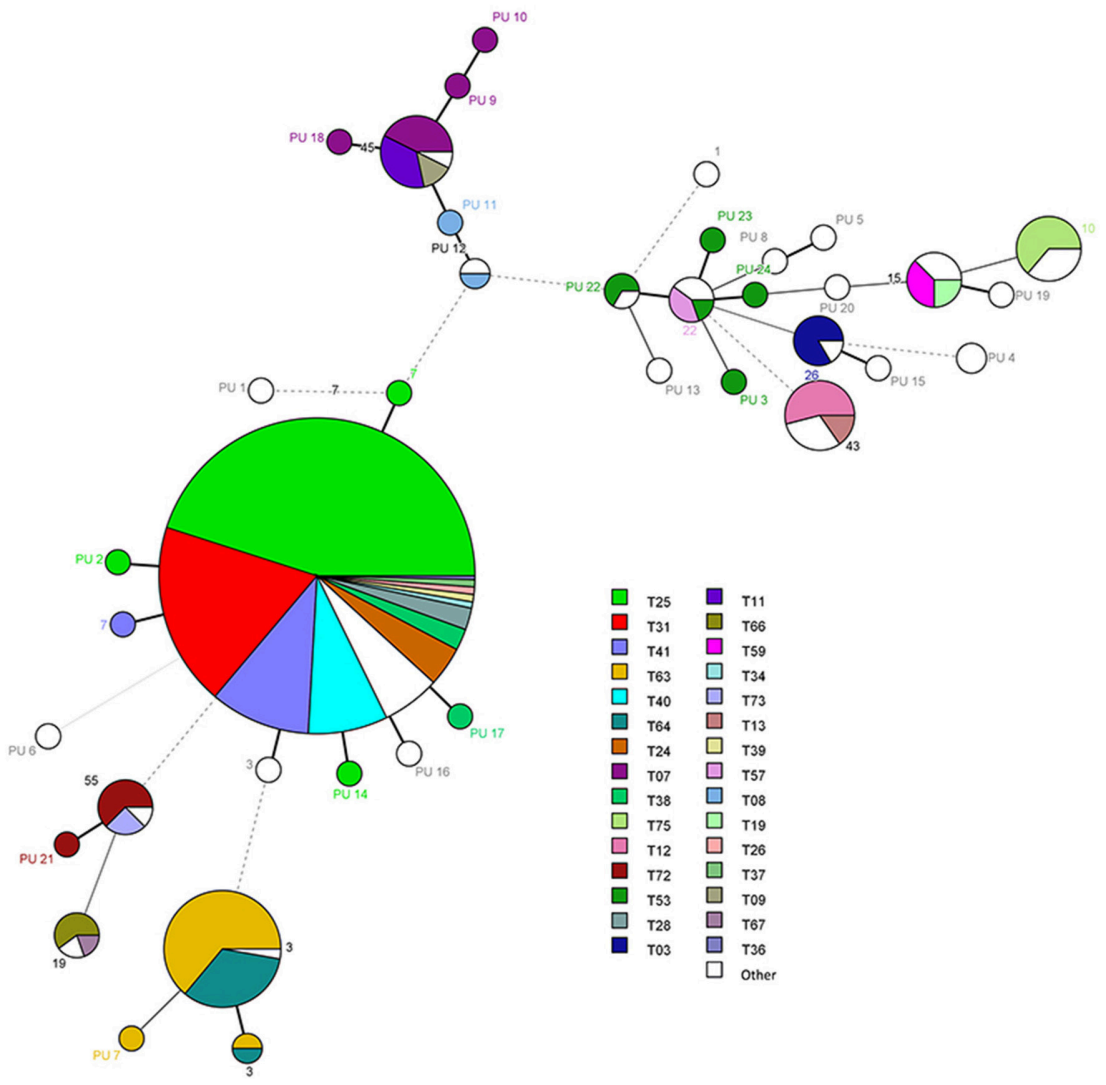
**Minimum spanning tree analysis based on allelic profiles of multilocus sequence typing (MLST)**. Each circle corresponds to a MLST ST. Different circle colors represent microsatellites genotypes.

Using microsatellite analyses, there were 79 genotypes amongst the 411 isolates designated as genotypes T01 to T79 (Table 2, Figures 2B, 3). Of the 79 genotypes, T25 (*n* = 125, 30.4%) and T31 (*n* = 51, 12.4%) were the most prevalent followed by genotype T41 (*n* = 29, 7.1%). T25 and T31 were the predominant genotypes in fluconazole susceptible-dose dependent (S-DD) as well as fluconazole-resistant isolates. Overall genotype distribution was similar for all clinical samples (Figures S3, S4). Twenty-four genotypes each comprised 2–25 isolates, with the remaining 52 genotypes comprising one isolate each. The diversity index varied from 0.46 for *RPM2* to 0.71 for *GLM4*. We found a D value of 0.88 (95% confidence interval: 0.86–0.90) by combining the six microsatellites (Table 1). Notably, there were 28 different microsatellite genotypes within ST7 (Figure 3), illustrating the higher D value of microsatellite-based polymorphism typing over MLST. The ST of *C. glabrata* was congruent (or consistent) with their microsatellite genotyping.

**Table 2:** **Designations of the 79 genotypes**.

**Genotype**	**Designation of microsatellite markers**						**No. (%) of isolates**
	**RPM2**	**ERG3**	**MTI**	**GLM4**	**GLM5**	**GLM6**	
T01	122	181	229	287	274	325	1 (0.2)
T02	122	181	237	287	274	325	1 (0.2)
T03	122	181	242	281	265	322	5 (1.2)
T04	122	181	242	284	265	322	1 (0.2)
T05	122	181	243	284	265	322	1 (0.2)
T06	122	234	243	266	262	247	1 (0.2)
T07	122	234	243	266	262	289	9 (2.2)
T08	122	234	243	266	265	289	2 (0.5)
T09	122	234	244	266	262	289	2 (0.5)
T10	122	234	244	266	265	289	1 (0.2)
T11	122	235	244	266	262	289	5 (1.2)
T12	122	238	242	293	262	310	7 (1.7)
T13	122	238	242	302	262	310	2 (0.5)
T14	122	238	242	305	262	310	1 (0.2)
T15	122	238	242	308	262	310	1 (0.2)
T16	122	238	243	293	262	310	1 (0.2)
T17	122	238	243	305	262	310	1 (0.2)
T18	128	181	229	278	268	325	1 (0.2)
T19	128	181	229	284	268	325	2 (0.5)
T20	128	181	243	287	262	307	1 (0.2)
T21	128	181	250	278	268	322	1 (0.2)
T22	128	197	241	272	298	295	1 (0.2)
T23	128	197	241	275	277	295	1 (0.2)
T24	128	197	241	275	295	295	11 (2.7)
T25	128	197	241	275	298	295	125 (30.4)
T26	128	197	241	275	301	295	2 (0.5)
T27	128	197	241	275	301	298	1 (0.2)
T28	128	197	241	275	304	295	6 (1.5)
T29	128	197	241	275	328	295	1 (0.2)
T30	128	197	241	275	331	295	1 (0.2)
T31	128	197	241	278	298	295	51 (12.4)
T32	128	197	241	278	298	301	1 (0.2)
T33	128	197	241	278	298	310	1 (0.2)
T34	128	197	241	278	301	295	2 (0.5)
T35	128	197	241	278	301	298	1 (0.2)
T36	128	197	241	278	304	295	1 (0.2)
T37	128	197	241	278	304	298	2 (0.5)
T38	128	197	241	278	304	301	7 (1.7)
T39	128	197	241	281	298	295	2 (0.5)
T40	128	197	242	278	298	295	22 (5.4)
T41	128	197	242	278	301	295	29 (7.1)
T42	128	197	242	278	304	307	1 (0.2)
T43	128	197	242	278	322	295	1 (0.2)
T44	128	197	242	278	331	295	1 (0.2)
T45	128	198	241	272	298	295	1 (0.2)
T46	128	198	241	275	295	295	1 (0.2)
T47	128	198	241	275	298	295	1 (0.2)
T48	128	198	241	275	304	295	1 (0.2)
T49	128	198	241	278	304	298	1 (0.2)
T50	128	198	242	275	301	295	1 (0.2)
T51	128	200	242	275	322	295	1 (0.2)
T52	128	213	229	287	268	325	1 (0.2)
T53	128	258	242	278	262	325	6 (1.5)
T54	128	262	242	278	259	325	1 (0.2)
T55	128	262	242	278	259	325	1 (0.2)
T56	128	262	243	278	265	325	1 (0.2)
T57	128	262	243	281	259	325	2 (0.2)
T58	134	181	229	275	268	325	1 (0.2)
T59	134	181	229	278	268	325	3 (0.2)
T60	134	181	229	281	268	325	1 (0.2)
T61	134	204	237	269	280	301	1 (0.2)
T62	134	204	243	269	262	310	1 (0.2)
T63	134	204	243	269	262	325	25 (6.1)
T64	134	204	244	269	262	325	13 (3.2)
T65	134	213	229	287	268	325	1 (0.2)
T66	134	213	241	281	265	298	3 (0.7)
T67	134	213	242	281	265	298	1 (0.2)
T68	134	213	242	284	265	298	1 (0.2)
T69	134	213	243	269	268	325	1 (0.2)
T70	134	235	241	284	268	325	1 (0.2)
T71	134	238	261	269	265	292	1 (0.2)
T72	140	197	241	275	265	295	6 (1.5)
T73	140	197	241	278	265	295	2 (0.5)
T74	140	197	242	278	265	295	1 (0.2)
T75	140	228	242	278	265	298	7 (1.7)
T76	140	228	243	281	265	298	1 (0.2)
T77	140	228	243	299	265	298	1 (0.2)
T78	140	230	242	278	265	298	1 (0.2)
T79	140	267	243	281	265	298	1 (0.2)

*The table shows the 79 genotypes (T01–T79) among 411 nonrepetitive Candida glabrata sensu stricto isolates generated by microsatellite genotyping*.

### Antifungal susceptibilities

The susceptibilities to antifungal drugs are shown in Table 3. Sixty-eight of 411 (16.5%) *C. glabrata* isolate*s* were resistant to fluconazole (MICs >32 μg/mL) (Clinical and Laboratory Standards Institute, 2012) with 40/200 (20%) of bloodstream isoaltes being resistant. The non-WT rates of *C. glabrata* for voriconazole, itraconazole and posaconazole were 28.7, 6.8, and 7.3% of isolates, respectively. Notably, 14.8% (61/411) of *C. glabrata* isolates were cross-resistant/non-WT to fluconazole and voriconazole. Caspofungin, micafungin and anidulafungin exhibited >99% susceptibility rates against all isolates. All isolates had WT MICs to amphotericin B. Only 0.2% (1/411) of *C. glabrata* isolates were non-WT to 5-flucytosine. The resistance rate for fluconazole increased significantly from 5.3% in 2013 to 31.4% in 2014 (P < 0.01) and non-WT rate for voriconazole increased significantly from 21.1% in 2013 to 82.6% in 2014 (*P* < 0.05). While the non-WT rate for itraconazole and posaconazole both decreased from 8.4% in 2012 to 1.3% in 2013 (*P* < 0.05) and remains 3.5% in 2014. There were no significant trends for resistance rate or non-WT rate for echinocandins, amphotericin B and 5-flucytosine (all the *P* > 0.05).

**Table 3:** **Antifungal susceptibilities for ***Candida glabrata*** species collected in this study**.

**Parameter**	**Fluconazole**	**Voriconazole**	**Itraconazole**	**Posaconazole**	**Anidulafungin**	**Micafungin**	**Caspofungin**	**5-flucytosine**	**Amphotericin B**
Range	1–>256	0.03–>8	0.12–>16	0.25–>8	≤0.015–>8	≤0.008–>8	≤0.008–>8	≤0.06–>64	≤0.12–2
MIC_90_	64	2	1	2	0.06	0.015	0.12	0.06	1
MIC_50_	16	0.25	0.5	1	0.03	0.015	0.06	0.06	0.5
0.78	19.29	0.36		1.20	0.028	0.013	0.073	0.062	0.65
R/non-WT (%)	16.5	28.7	6.8	7.3	0.5	0.5	0.5	0.2	0

*GM, geometric mean; R, resistant; non-WT, non-wide-type*.

## Discussion

*Candida glabrata* is an increasingly important pathogen in the United States and Europe but also in China (Pfaller et al., 2001; Wang et al., 2012; Guinea, 2014; Xiao et al., 2015; Delliere et al., 2016). Knowledge of both the diversity of molecular types, as well as antifungal susceptibility profiles of *C. glabrata* are important for understanding the epidemiology of this organism. Our study, for the first time, provides a description of the genetic diversity and antifungal susceptibility of a large number of *C. glabrata* strains. Major findings of the study included the observations that fluconazole resistant rates increased 3-fold over the 5-year period, the frequency of isolates with non-WT MICs to voriconazole rose 7-fold, and that Chinese *C. glabrata* sensu stricto isolates exhibit relatively low intraspecies genetic diversity.

Despite the position of *C. glabrata* as a pathogen in China, we noted that the isolation rate of *C. glabrata* slightly decreased in 2013 from 2010. The reason for this apparent drop may be because of a large-scale outbreak of *Candida parapsilosis* sensu stricto fungemia involving >100 isolates in one of the 11 participating hospitals during the study period (Wang et al., 2016). Nonetheless *C. glabrata* accounted for 10.2% (200/1963) of candidemia cases in the present study, similar to that found in Finland (9.0%) and Norway (13.2%) (Guinea, 2014) but substantially less than that in Denmark (25%) and the USA (21%) (Pfaller et al., 2001; Arendrup et al., 2011). In this study, most of isolates were recovered from blood (200/411; 48.7%), while in a French study, most of isolates were collected from respiratory sample (81/268; 30.2%) (Delliere et al., 2016).

Strain typing is essential for epidemiological investigation and a variety of molecular methods have been applied for genotyping of *C. glabrata*. PFGE exhibits high discriminatory power, but is limited by the high initial investment costs and slow turn-around times (Abbes et al., 2010). MLST has the advantage of providing unambiguous results, which allows different laboratories to easily compare data and allows for the construction of international internet-accessible databases (Dodgson et al., 2003). However, the D value was only 0.55 in the present study. It has been reported that MLST system developed for *C. glabrata* appears to be less discriminatory than that for *C. albicans*. One plausible explanatio*n* is that *C. albicans* is a diploid organism, as opposed to the haploid status of *C. glabrata*, which allows for greater potential for detecting the presence of genetic heterogeneity with the former (Dodgson et al., 2003). As such, we found a low degree of genetic diversity amongst *C. glabrata* using MLST analysis. The majority (75.9%) of isolates comprised only two STs, ST7 and ST3. *C. glabrata* ST3 and ST5 types have been predominant in Europe, while ST7 and ST30 types are reported to be the most common in Japan (Dodgson et al., 2003), and ST8 and ST18 types in the USA (Dodgson et al., 2003). The differences in STs according to geography, highlight the significance of acquiring local data.

The results of the present study show that in comparison to MLST, the *D*-value for microsatellite typing was 0.88, higher than MLST employed herein, but lower than that in one study using microsatellite analysis (Abbes et al., 2012). Nonetheless, the results of microsatellite genotyping in our study were concordant with those of a predominant genotype identified. Our isolates were collected only from Chinese patients and it is logical that coevolution of genetic markers will provide similar results by any chose typing method. The *D*-value may be improved by incorporation of a greater number of more loci by the microsatellite analysis approach and this is the focus of ongoing study (Dodgson et al., 2003; Foulet et al., 2005; Abbes et al., 2012; Delliere et al., 2016). This approach is also simple to use and is inexpensive (US$9 per sample analyzed vs. US$24 for MLST).

Of note, there was no correlation between genetic type and isolates from patients at the different hospitals or from departments by either MLST or microsatellite typing. We also found no association between genetic type and susceptibility to fluconazole (Tables S1, S2, Figures S1, S3). However, our results do not exclude the possibility that certain STs or microsatellite genotypes may have the capacity to acquire resistance through drug exposure at differing frequencies. Many studies have likewise found no association between *C. glabrata* genotypes and antifungal resistance (Dodgson et al., 2003; Abbes et al., 2011). However, Dhieb et al. noted that both microsatellite genotypes and MALDI-TOF MS analysis could highlight *C. glabrata* population structures associated with specific geographic origin or antifungal drug resistance pattern (Dhieb et al., 2015). In our study, we noticed that during 2010–2011 in one hospital (Hospital BD) 80.8% (21/26) *C. glabrata* isolates were of the same genetic type (ST7 and T25), suggesting possible clonal presence/transmission of *C. glabrata*. 14 isolates were from blood, four from ascitic fluid, two from venous catheter and one from bronchoalveolar lavage fluid, and five of these isolates (5/21, 23.8%) were collected from patients in the same department and were resistant to fluconazole. Further study is needed to investigate the clinical events at this hospital stemming from this observation.

Importantly, our results show that only 6.8% of *C. glabrata* isolates were non-WT/resistant to all four azoles tested in contrast to results noted in the USA (Pfaller et al., 2004), but comparable to those reported by Wang et al. (2012) and Delliere et al. (2016). Of note, however, the proportion of isolates that were fluconazole–resistant and/or had non-WT MICs to voriconazole rose significantly over 5 years. That many of these isolates remained susceptible to posaconazole and itraconazole underscores the importance of susceptibility testing for individual isolates.

Echinocandins have become the first-line treatment of IC caused by *C. glabrata* (Pfaller et al., 2012). In this context, the fact that only two isolates (0.5% of all isolates) tested resistant to the echinocandins (Clinical and Laboratory Standards Institute, 2012) is reassuring. One *C. glabrata* isolate was observed to have an MIC > 8 μg/mL to all three echinocandins. The low rate of echinocandin resistance here contrasts with that in the USA and elsewhere where resistance has bene reported in up to 10% and with one-third of those isolates being multidrug resistant (Pfaller et al., 2012; Alexander et al., 2013; Eschenauer et al., 2014; Pham et al., 2014). However, one French study observed only a low proportion of isolates to be resistant to micafungin (0.7%) using the Etest (bioMérieux, Marcy l'Etoile, France) and employing European Committee on Antimicrobial Susceptibility Testing (EUCAST) breakpoints, with only 1/268 isolates showing cross-resistance to both antifungal classes (Delliere et al., 2016). Importantly, one echinocandin-resistant isolate in the present study was also resistant to fluconazole, which MICs for fluconazole, voriconazole, itraconazole and posaconazole were 128, 8, ≥16, and ≥8 μg/mL, respectively and 0.5 μg/mL for echinocandins. This is the first multi-drug resistant isolate reported in China. Elsewhere, about 11.1% of fluconazole-resistant *C. glabrata* were co-resistant to one or more echinocandins study (Pfaller et al., 2012).

One study limitation is that we used the SYO methodology to perform antifungal susceptibility testing. The essential agreement between this methodology and the CLSI as well as with the EUCAST reference procedures are known to be very high (Cuenca-Estrella and Rodriguez-Tudela, 2010; Posteraro and Sanguinetti, 2014). In addition, the Sensititre method is a simple and affordable alternative to these reference methodologies and is widely used in clinical mycology laboratories (Posteraro and Sanguinetti, 2014).

## Conclusion

This is the first systemic study regarding the molecular epidemiology and antifungal susceptibility profiles of *C. glabrata* isolates in China. Identification of relatedness between *C. glabrata* is important in understanding their molecular epidemiology. Our results suggest that some *C. glabrata* populations are more prominent than others. Further investigations are needed to confirm this hypothesis.

## Author contributions

XH, MX, YZ, and YX conceived and designed the research; XH, GZ, HW, and XF collaborated in molecular investigations of the strains; HW, SC, FK, YC, MK, ZS, ZH, RL, JL, KL, TH, YN, and G-LZ provided yeasts and analyzed the data; XH, MX, SC, and FK wrote the manuscript. All authors read, improved and reviewed the manuscript.

## Funding

This work was supported by the Research Special Fund for Public Welfare Industry of Health (No. 201402001), the Innovation Fund of Peking Union Medical College (No. 2016-1001-15) and CAMS Innovation Fund for Medical Sciences (No. 2016-I2M-1-014). Funding sources had no role in the study design, data collection and analysis, decisions to publish, or preparation of the manuscript.

### Conflict of interest statement

The authors declare that the research was conducted in the absence of any commercial or financial relationships that could be construed as a potential conflict of interest.

## Supplementary material

Figure S1 **Minimum spanning tree analysis based on allelic profiles of multilocus sequence typing (MLST)**. Different circle colors represent fluconazole susceptible-dose dependent (S-DD) (green) or resistant (MIC > 32 μg/ML) (red). Each circle in corresponds to a MLST ST.

Figure S2 **Minimum spanning tree analysis based on allelic profiles of multilocus sequence typing (MLST)**. Different circle colors represent sample types. Each circle in corresponds to a MLST ST.

Figure S3 **Minimum spanning tree analysis based on allelic profiles of microsatellites genotypes**. Different circle colors represent fluconazole susceptible-dose dependent (S-DD) (green) or resistant (MIC > 32 μg/ML) (red). Each circle in corresponds to a microsatellites genotype.

Figure S4 **Minimum spanning tree analysis based on allelic profiles of microsatellites genotypes**. Different circle colors represent sample types. Each circle in corresponds to a microsatellites genotype.

Table S1 **Distribution of ***Candida glabrata*** isolates of different sequence types (STs) in different specimen types and prevalence of fluconazole resistant (MIC > 32 μg/mL) isolates**.

Table S2 **Distribution of ***Candida glabrata*** isolates of different microsatellite genotypes in different specimen types and prevalence of fluconazole resistant (MIC > 32 μg/mL) isolates**.
