# Supplementary figures and images for: Molecular Epidemiology and Antifungal Susceptibility of Candida glabrata in China (August 2009 to July 2014): A Multi-Center Study
Source: Front Microbiol. 2017 May 23;8:880. doi: 10.3389/fmicb.2017.00880 (PMC5440528; doi:10.3389/fmicb.2017.00880)

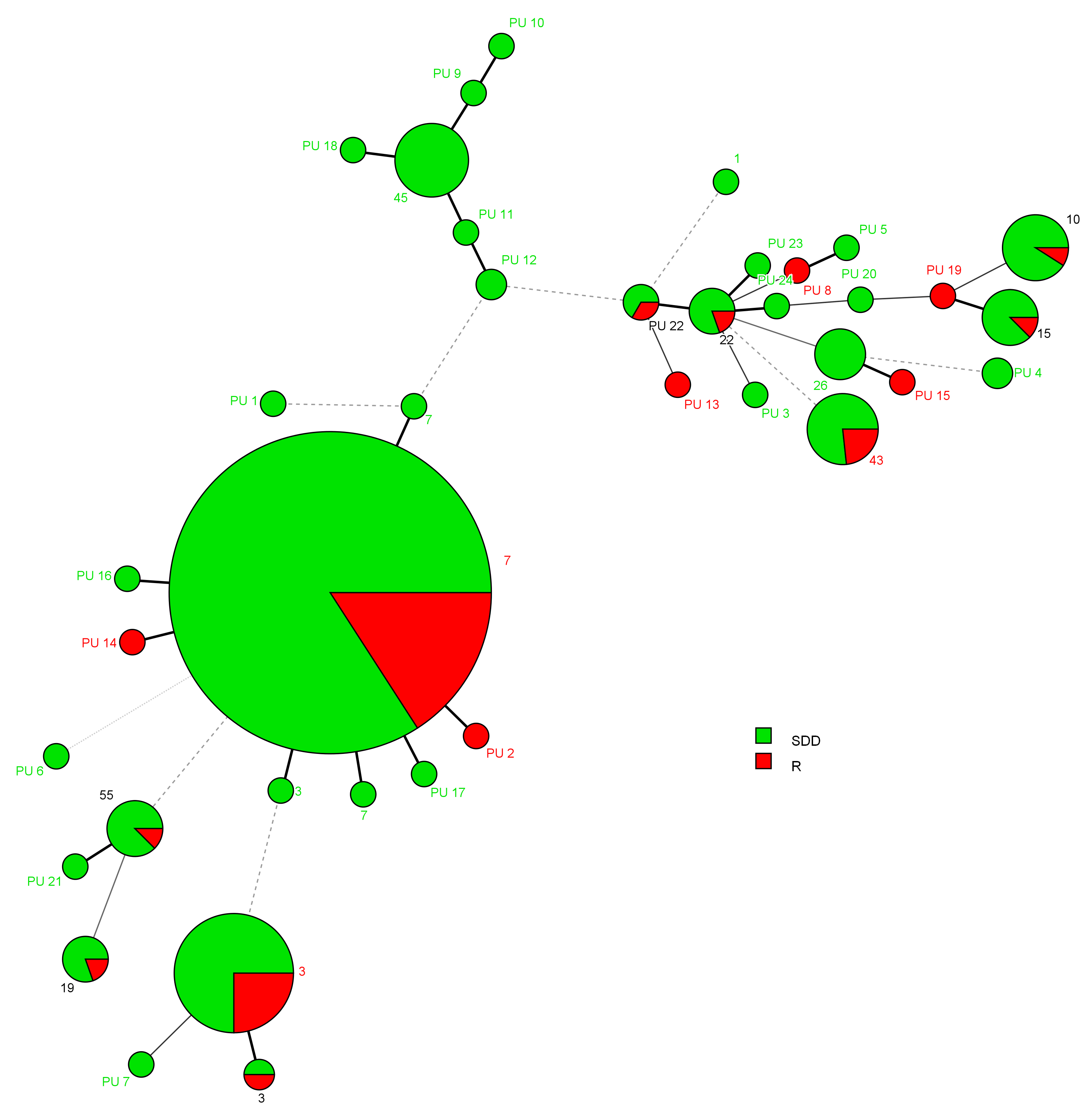

Supplement: Figure S1 — Minimum spanning tree analysis based on allelic profiles of multilocus sequence typing (MLST). Different circle colors represent fluconazole susceptible-dose dependent (S-DD) (green) or resistant (MIC > 32 μg/ML) (red). Each circle in corresponds to a MLST ST. [file Image1.jpg]

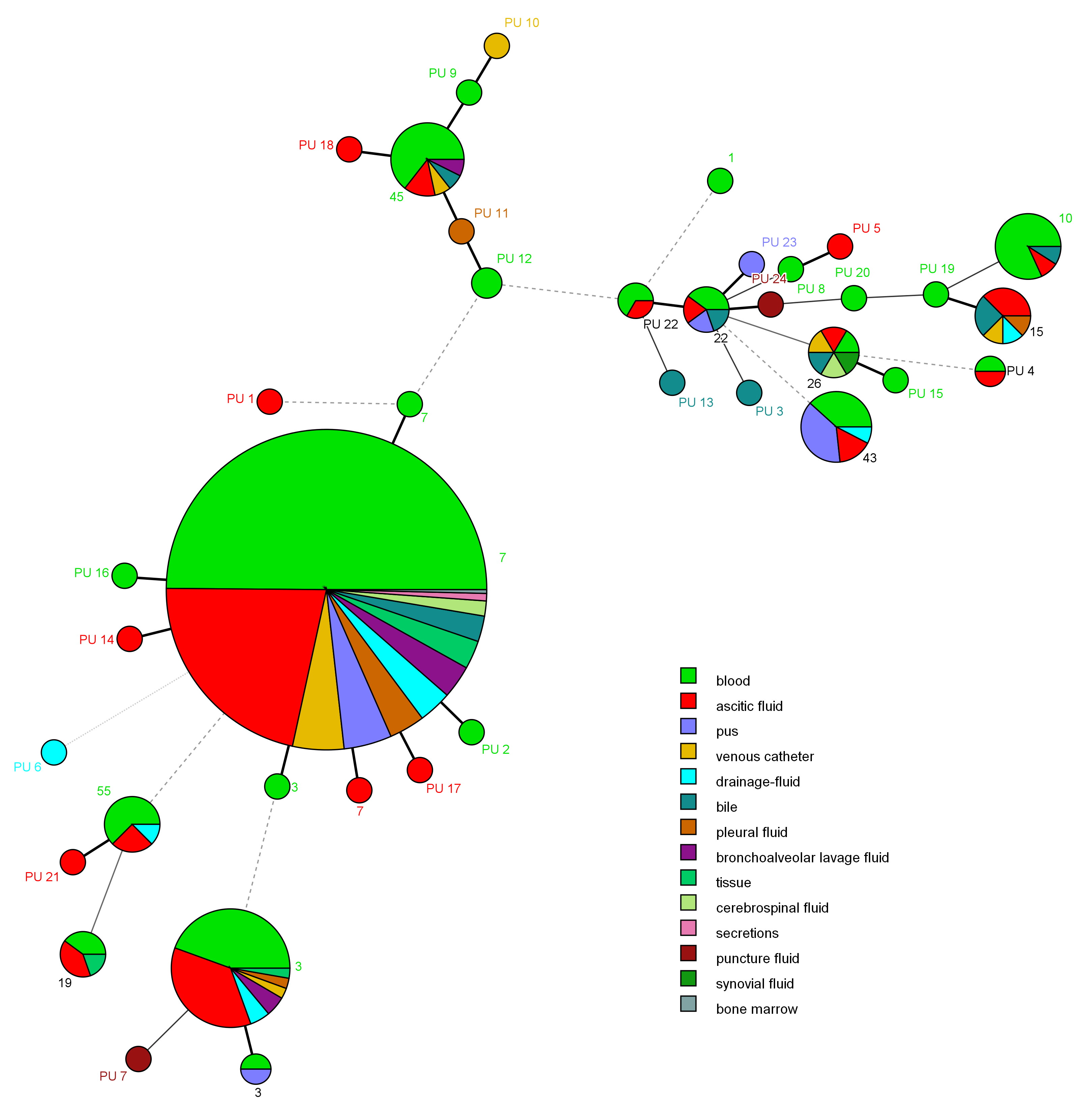

Supplement: Figure S2 — Minimum spanning tree analysis based on allelic profiles of multilocus sequence typing (MLST). Different circle colors represent sample types. Each circle in corresponds to a MLST ST. [file Image2.jpg]

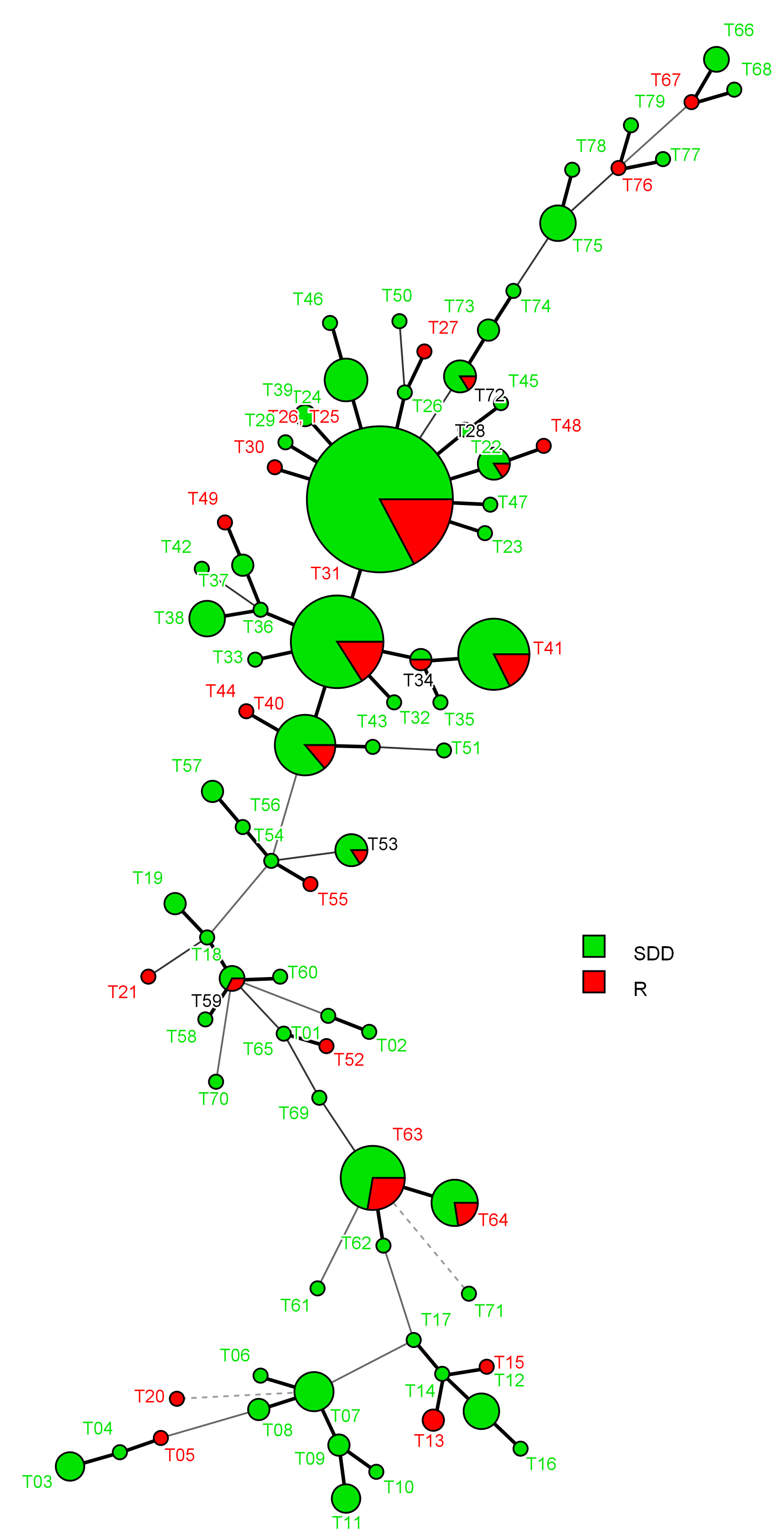

Supplement: Figure S3 — Minimum spanning tree analysis based on allelic profiles of microsatellites genotypes. Different circle colors represent fluconazole susceptible-dose dependent (S-DD) (green) or resistant (MIC > 32 μg/ML) (red). Each circle in corresponds to a microsatellites genotype. [file Image3.jpg]

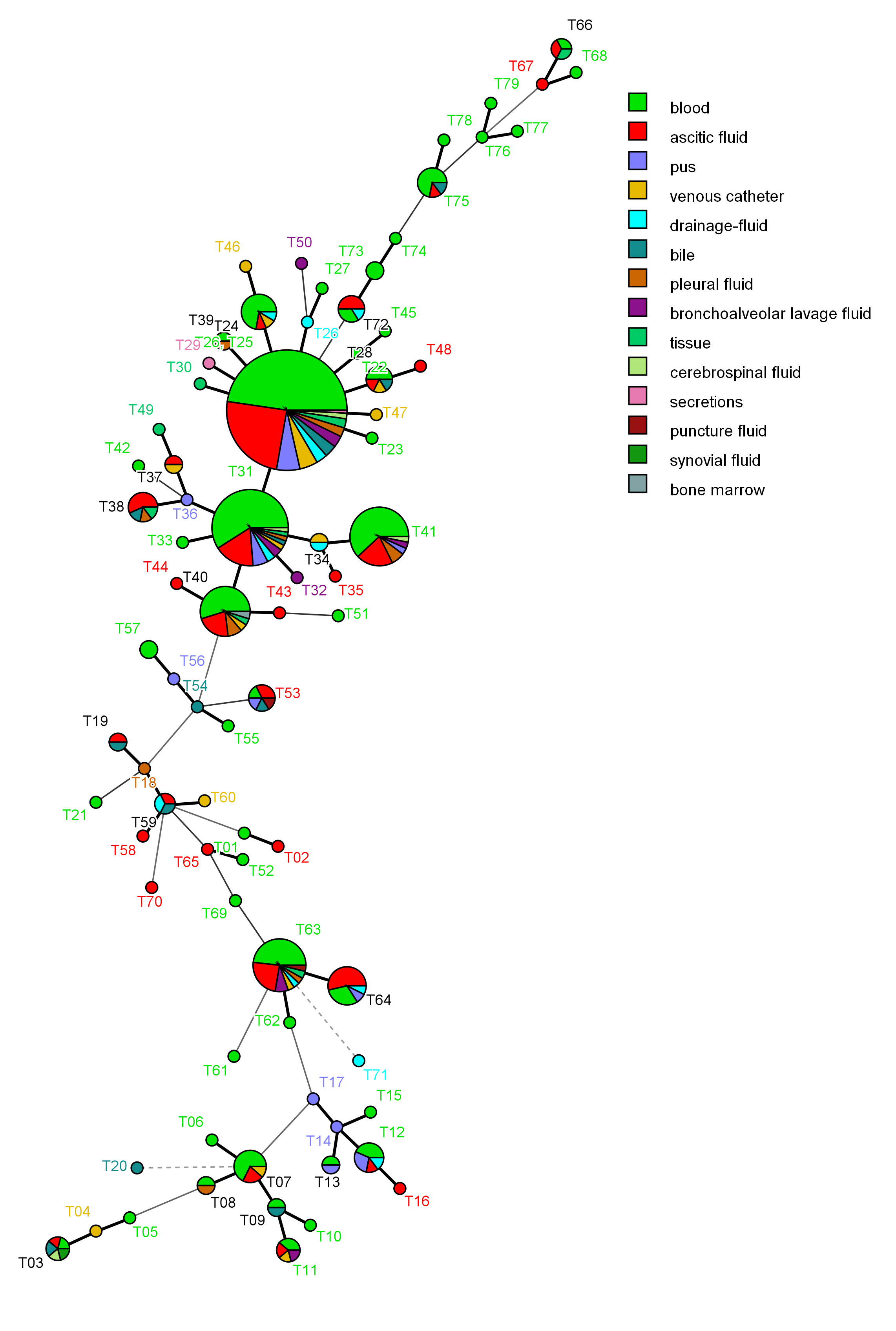

Supplement: Figure S4 — Minimum spanning tree analysis based on allelic profiles of microsatellites genotypes. Different circle colors represent sample types. Each circle in corresponds to a microsatellites genotype. [file Image4.jpg]
