# Supplementary material for: Molecular Epidemiology and Antifungal Susceptibility of Candida glabrata in China (August 2009 to July 2014): A Multi-Center Study
Source: Front Microbiol. 2017 May 23;8:880. doi: 10.3389/fmicb.2017.00880 (PMC5440528; doi:10.3389/fmicb.2017.00880)
Supplement: Table S1 — Distribution of Candida glabrata isolates of different sequence types (STs) in different specimen types and prevalence of fluconazole resistant (MIC > 32 μg/mL) isolates. [file Table1.docx]

**Table S1 Distribution of *Candida glabrata* isolates of different sequence types (STs) in different specimen types and prevalence of fluconazole resistant (MIC > 32μg/mL) isolates**

|  | **No. of *C. glabrata* isolates (% of total)** | | | | | |
| --- | --- | --- | --- | --- | --- | --- |
|  | **Specimen type** | | | |  | |
| **Sequence type (ST)** | **Blood** | **Ascitic fluid** | **Other specimen types** | | **Resistant to fluconazole** | |
| 7 | 136 (68.0) | 60 (63.2) | 77 (66.4) | 43 (63.2) | |  |
| 3 | 18 (9.0) | 13 (13.7) | 8 (6.9) | 10 (14.7) | |  |
| 45 | 9 (4.5) | 2 (2.1) | 3 (2.6) | 0 (0.0) | |  |
| 43 | 5 (2.5) | 2 (2.1) | 6 (5.2) | 3 (4.4) | |  |
| 10 | 9 (4.5) | 1 (1.1) | 1 (0.9) | 1 (1.5) | |  |
| 15 | 0 (0.0) | 3 (3.2) | 5 (4.3) | 1 (1.5) | |  |
| 55 | 5 (2.5) | 2 (2.1) | 1 (0.9) | 1 (1.5) | |  |
| 26 | 1 (0.5) | 1 (1.1) | 4 (3.4) | 0 (0.0) | |  |
| 19 | 2 (1.0) | 2 (2.1) | 1 (0.9) | 1 (1.5) | |  |
| 22 | 2 (1.0) | 1 (1.1) | 2 (1.7) | 1 (1.5) | |  |
| 1 | 1 (0.5) | 0 (0.0) | 0 (0.0) | 0 (0.0) | |  |
| PU 22 | 2 (1.0) | 1 (1.1) | 0 (0.0) | 1 (1.5) | |  |
| PU 12 | 2 (1.0) | 0 (0.0) | 0 (0.0) | 0 (0.0) | |  |
| PU 4 | 1 (0.5) | 1 (1.1) | 0 (0.0) | 0 (0.0) | |  |
| PU 1 | 0 (0.0) | 1 (1.1) | 0 (0.0) | 0 (0.0) | |  |
| PU 2 | 1 (0.5) | 0 (0.0) | 0 (0.0) | 1 (1.5) | |  |
| PU 3 | 0 (0.0) | 0 (0.0) | 1 (0.9) | 0 (0.0) | |  |
| PU 5 | 0 (0.0) | 1 (1.1) | 0 (0.0) | 0 (0.0) | |  |
| PU 6 | 0 (0.0) | 0 (0.0) | 1 (0.9) | 0 (0.0) | |  |
| PU 7 | 0 (0.0) | 0 (0.0) | 1 (0.9) | 0 (0.0) | |  |
| PU 8 | 1 (0.5) | 0 (0.0) | 0 (0.0) | 1 (1.5) | |  |
| PU 9 | 1 (0.5) | 0 (0.0) | 0 (0.0) | 0 (0.0) | |  |
| PU 10 | 0 (0.0) | 0 (0.0) | 1 (0.9) | 0 (0.0) | |  |
| PU 11 | 0 (0.0) | 0 (0.0) | 1 (0.9) | 0 (0.0) | |  |
| PU 13 | 0 (0.0) | 0 (0.0) | 1 (0.9) | 1 (1.5) | |  |
| PU 14 | 0 (0.0) | 1 (1.1) | 0 (0.0) | 1 (1.5) | |  |
| PU 15 | 1 (0.5) | 0 (0.0) | 0 (0.0) | 1 (1.5) | |  |
| PU 16 | 1 (0.5) | 0 (0.0) | 0 (0.0) | 0 (0.0) | |  |
| PU 17 | 0 (0.0) | 1 (1.1) | 0 (0.0) | 0 (0.0) | |  |
| PU 18 | 0 (0.0) | 1 (1.1) | 0 (0.0) | 0 (0.0) | |  |
| PU 19 | 1 (0.5) | 0 (0.0) | 0 (0.0) | 1 (1.5) | |  |
| PU 20 | 1 (0.5) | 0 (0.0) | 0 (0.0) | 0 (0.0) | |  |
| PU 21 | 0 (0.0) | 1 (1.1) | 0 (0.0) | 0 (0.0) | |  |
| PU 23 | 0 (0.0) | 0 (0.0) | 1 (0.9) | 0 (0.0) | |  |
| PU 24 | 0 (0.0) | 0 (0.0) | 1 (0.9) | 0 (0.0) | |  |
| Total | 200 (100) | 95 (100) | 116 (100) | 68 (100) | |  |
