# Supplementary material for: Molecular Epidemiology and Antifungal Susceptibility of Candida glabrata in China (August 2009 to July 2014): A Multi-Center Study
Source: Front Microbiol. 2017 May 23;8:880. doi: 10.3389/fmicb.2017.00880 (PMC5440528; doi:10.3389/fmicb.2017.00880)
Supplement: Table S2 — Distribution of Candida glabrata isolates of different microsatellite genotypes in different specimen types and prevalence of fluconazole resistant (MIC > 32 μg/mL) isolates. [file Table2.docx]

**Table S2 Distribution of *Candida glabrata* isolates of different microsatellite genotypes in different specimen types and prevalence of fluconazole resistant (MIC > 32μg/mL) isolates**

|  | **No. of *C. glabrata* isolates (% of total)** | | | |
| --- | --- | --- | --- | --- |
|  | **Specimen type** | | |  |
| **Genotype** | **Blood** | **Ascetic fluid** | **Other specimen types** | **Resistant to fluconazole** |
| T25 | 60 (30.0) | 31 (32.6) | 34 (29.3) | 22 (32.4) |
| T31 | 30 (15.0) | 9 (9.5) | 12 (10.3) | 8 (11.8) |
| T41 | 18 (9.0) | 6 (6.3) | 5 (4.3) | 5 (7.4) |
| T63 | 12 (6.0) | 6 (6.3) | 7 (6.0) | 7 (10.3) |
| T40 | 12 (6.0) | 5 (5.3) | 5 (4.3) | 3 (4.4) |
| T64 | 4 (2.0) | 7 (7.4) | 2 (1.7) | 3 (4.4) |
| T24 | 8 (4.0) | 1 (1.1) | 2 (1.7) | 0 (0.0) |
| T07 | 6 (3.0) | 2 (2.1) | 1 (0.9) | 0 (0.0) |
| T12 | 3 (1.5) | 1 (1.1) | 3 (2.6) | 0 (0.0) |
| T38 | 0 (0.0) | 4 (4.2) | 3 (2.6) | 0 (0.0) |
| T75 | 5 (2.5) | 1 (1.1) | 1 (0.9) | 0 (0.0) |
| T28 | 3 (1.5) | 1 (1.1) | 2 (1.7) | 1 (1.5) |
| T53 | 1 (0.5) | 2 (2.1) | 3 (2.6) | 1 (1.5) |
| T72 | 2 (1.0) | 3 (3.2) | 1 (0.9) | 1 (1.5) |
| T03 | 1 (0.5) | 1 (1.1) | 3 (2.6) | 0 (0.0) |
| T11 | 2 (1.0) | 1 (1.1) | 2 (1.7) | 0 (0.0) |
| T59 | 0 (0.0) | 1 (1.1) | 2 (1.7) | 1 (1.5) |
| T66 | 1 (0.5) | 1 (1.1) | 1 (0.9) | 0 (0.0) |
| T08 | 1 (0.5) | 0 (0.0) | 1 (0.9) | 0 (0.0) |
| T09 | 1 (0.5) | 0 (0.0) | 1 (0.9) | 0 (0.0) |
| T13 | 1 (0.5) | 0 (0.0) | 1 (0.9) | 2 (2.9) |
| T19 | 0 (0.0) | 1 (1.1) | 1 (0.9) | 0 (0.0) |
| T26 | 0 (0.0) | 0 (0.0) | 2 (1.7) | 0 (0.0) |
| T34 | 0 (0.0) | 0 (0.0) | 2 (1.7) | 1 (1.5) |
| T37 | 0 (0.0) | 1 (1.1) | 1 (0.9) | 0 (0.0) |
| T39 | 1 (0.5) | 0 (0.0) | 1 (0.9) | 0 (0.0) |
| T57 | 2 (1.0) | 0 (0.0) | 0 (0.0) | 0 (0.0) |
| T73 | 2 (1.0) | 0 (0.0) | 0 (0.0) | 0 (0.0) |
| T01 | 1 (0.5) | 0 (0.0) | 0 (0.0) | 0 (0.0) |
| T02 | 0 (0.0) | 1 (1.1) | 0 (0.0) | 0 (0.0) |
| T04 | 0 (0.0) | 0 (0.0) | 1 (0.9) | 0 (0.0) |
| T05 | 1 (0.5) | 0 (0.0) | 0 (0.0) | 1 (1.5) |
| T06 | 1 (0.5) | 0 (0.0) | 0 (0.0) | 0 (0.0) |
| T10 | 1 (0.5) | 0 (0.0) | 0 (0.0) | 0 (0.0) |
| T14 | 0 (0.0) | 0 (0.0) | 1 (0.9) | 0 (0.0) |
| T15 | 1 (0.5) | 0 (0.0) | 0 (0.0) | 1 (1.5) |
| T16 | 0 (0.0) | 1 (1.1) | 0 (0.0) | 0 (0.0) |
| T17 | 0 (0.0) | 0 (0.0) | 1 (0.9) | 0 (0.0) |
| T18 | 0 (0.0) | 0 (0.0) | 1 (0.9) | 0 (0.0) |
| T20 | 0 (0.0) | 0 (0.0) | 1 (0.9) | 1 (1.5) |
| T21 | 1 (0.5) | 0 (0.0) | 0 (0.0) | 1 (1.5) |
| T22 | 1 (0.5) | 0 (0.0) | 0 (0.0) | 0 (0.0) |
| T23 | 1 (0.5) | 0 (0.0) | 0 (0.0) | 0 (0.0) |
| T27 | 1 (0.5) | 0 (0.0) | 0 (0.0) | 1 (1.5) |
| T29 | 0 (0.0) | 0 (0.0) | 1 (0.9) | 0 (0.0) |
| T30 | 0 (0.0) | 0 (0.0) | 1 (0.9) | 1 (1.5) |
| T32 | 0 (0.0) | 0 (0.0) | 1 (0.9) | 0 (0.0) |
| T33 | 1 (0.5) | 0 (0.0) | 0 (0.0) | 0 (0.0) |
| T35 | 0 (0.0) | 1 (1.1) | 0 (0.0) | 0 (0.0) |
| T36 | 0 (0.0) | 0 (0.0) | 1 (0.9) | 0 (0.0) |
| T42 | 1 (0.5) | 0 (0.0) | 0 (0.0) | 0 (0.0) |
| T43 | 0 (0.0) | 1 (1.1) | 0 (0.0) | 0 (0.0) |
| T44 | 0 (0.0) | 1 (1.1) | 0 (0.0) | 1 (1.5) |
| T45 | 1 (0.5) | 0 (0.0) | 0 (0.0) | 0 (0.0) |
| T46 | 0 (0.0) | 0 (0.0) | 1 (0.9) | 0 (0.0) |
| T47 | 0 (0.0) | 0 (0.0) | 1 (0.9) | 0 (0.0) |
| T48 | 0 (0.0) | 1 (1.1) | 0 (0.0) | 1 (1.5) |
| T49 | 0 (0.0) | 0 (0.0) | 1 (0.9) | 1 (1.5) |
| T50 | 0 (0.0) | 0 (0.0) | 1 (0.9) | 0 (0.0) |
| T51 | 1 (0.5) | 0 (0.0) | 0 (0.0) | 0 (0.0) |
| T52 | 1 (0.5) | 0 (0.0) | 0 (0.0) | 1 (1.5) |
| T54 | 0 (0.0) | 0 (0.0) | 1 (0.9) | 0 (0.0) |
| T55 | 1 (0.5) | 0 (0.0) | 0 (0.0) | 1 (1.5) |
| T56 | 0 (0.0) | 0 (0.0) | 1 (0.9) | 0 (0.0) |
| T58 | 0 (0.0) | 1 (1.1) | 0 (0.0) | 0 (0.0) |
| T60 | 0 (0.0) | 0 (0.0) | 1 (0.9) | 0 (0.0) |
| T61 | 1 (0.5) | 0 (0.0) | 0 (0.0) | 0 (0.0) |
| T62 | 1 (0.5) | 0 (0.0) | 0 (0.0) | 0 (0.0) |
| T65 | 0 (0.0) | 1 (1.1) | 0 (0.0) | 0 (0.0) |
| T67 | 0 (0.0) | 1 (1.1) | 0 (0.0) | 1 (1.5) |
| T68 | 1 (0.5) | 0 (0.0) | 0 (0.0) | 0 (0.0) |
| T69 | 1 (0.5) | 0 (0.0) | 0 (0.0) | 0 (0.0) |
| T70 | 0 (0.0) | 1 (1.1) | 0 (0.0) | 0 (0.0) |
| T71 | 0 (0.0) | 0 (0.0) | 1 (0.9) | 0 (0.0) |
| T74 | 1 (0.5) | 0 (0.0) | 0 (0.0) | 0 (0.0) |
| T76 | 1 (0.5) | 0 (0.0) | 0 (0.0) | 1 (1.5) |
| T77 | 1 (0.5) | 0 (0.0) | 0 (0.0) | 0 (0.0) |
| T78 | 1 (0.5) | 0 (0.0) | 0 (0.0) | 0 (0.0) |
| T79 | 1 (0.5) | 0 (0.0) | 0 (0.0) | 0 (0.0) |
| Total | 200 (100) | 95 (100) | 116 (100) | 68 (100) |
